# Number of tumor foci predicts prognosis in papillary thyroid cancer

**DOI:** 10.1186/1471-2407-14-914

**Published:** 2014-12-04

**Authors:** Ning Qu, Ling Zhang, Qing-hai Ji, Yong-xue Zhu, Zhuo-ying Wang, Qiang Shen, Yu Wang, Duan-shu Li

**Affiliations:** Department of Head and Neck Surgery, Fudan University Shanghai Cancer Center; Department of Oncology, Shanghai Medical College, Fudan University, Shanghai, 200032 China

**Keywords:** Papillary thyroid carcinoma, Multifocality, Recurrence, Mortality

## Abstract

**Background:**

Papillary thyroid cancer (PTC) often presents as multifocal. However, the association of multifocality with poor prognosis remains controversial. The aim of this retrospective study was to identify the characteristics of PTC with multiple foci and to evaluate the association between multifocality and prognosis.

**Methods:**

We reviewed the medical records of 496 patients who underwent total thyroidectomy for PTC. Patients were classified as G1 (1 tumor focus), G2 (2 foci), and G3 (3 or more foci). We analyzed the clinicopathological features and clinical outcomes in each classification. A Cox regression model was used to assess the relationship between multifocality and recurrence or cancer mortality.

**Results:**

The G1, G2 and G3 groups included 287, 141 and 68 patients, respectively. The mean age was 47.1 ± 16.1 yr in G1, 41.1 ± 18.4 yr in G2, and 35.5 ± 15.9 yr in G3 and differed significantly among the 3 groups (*p* = 0.001). The proportion of extrathyroidal extension, central lymph node metastasis (CLNM), and lateral lymph node metastasis (LLNM) in the G1 to G3 groups increased with increasing number of tumor foci. The Kaplan–Meier curves revealed that G3 had the shortest recurrence-free survival, and differences were significant among the 3 groups (*p* = 0.001, Log Rank test). Furthermore, cancer-specific survival rates decreased significantly with increasing number of tumor foci (*p* = 0.041). Independent predictors of recurrence by multivariate Cox analysis included >3 tumor foci [HR 2.60, 95% confidence interval (CI) 1.53-4.39, *p* = 0.001] and extrathyroidal extension (HR 1.95, CI 1.12-3.38, *p* = 0.018).

**Conclusion:**

An increase in the number of tumors is associated with a tendency toward more aggressive features and predicts poor prognosis in PTC.

## Background

The number of newly diagnosed thyroid carcinoma cases is increasing annually, and an estimated 62,980 cases will be diagnosed in the United States in 2014, more than 90% of which will be papillary thyroid cancer (PTC) [1, 2]. PTC often presents as multifocal tumors [3], which are thought to arise independently, indicating an inherent propensity to develop PTC, and spread throughout the thyroid gland [4].

PTC is generally an indolent disease and has a favorable prognosis in most affected patients; however, PTC with multifocal foci is likely to be aggressive and, accordingly, require aggressive treatment [5, 6]. The characteristics of PTC with multifocal foci and the prognostic significance of multifocality in PTC remain controversial [7, 8]. We performed a retrospective analysis to examine the characteristics of multifocal PTC and the relationship between the number of tumor foci and PTC prognosis in a large group of Chinese patients with long-term follow-up.

## Methods

### Patients

The records of all PTC patients treated at our center between January 1, 1983, and December 31, 2007, were retrospectively reviewed. All patients provided written informed consent for their information to be stored in the hospital database and used for research, and this study was approved by the Ethical Committee of the Cancer Center of Fudan University. A series of 496 consecutive PTC patients who underwent primary surgical therapy of total thyroidectomy (TT) were enrolled in this study, representing 23.5% of the total 2115 patients treated at our center during the corresponding period.

### Initial treatment

Before surgery, each patient underwent an ultrasonography (US) examination. US-guided fine-needle aspiration (FNA) was not performed routinely. Lobectomy plus ipsilateral central lymph node dissection (CLND) was typically performed as the initial surgical treatment for PTC patients with malignant lesions that were limited to a single lobe [9]. When the patient was older than 45 yr, the primary tumor was greater than 1 cm, undetermined nodules were detected in the contralateral lobe by US or regional metastases or multifocal tumors were present, TT was performed at the time of the initial operation. When undetermined nodules were detected in the contralateral lobe by US without other factors, a subtotal lobectomy of approximately one-fourth to two-thirds of the contralateral lobe was performed on the suspicion of lesions in the contralateral lobe following the preoperative US. Histology of the frozen sections (FS) assisted surgeons in determining the extent of the surgical procedures. When malignant lesions were identified in both lobes of the thyroid by FS after a subtotal lobectomy, a completion thyroidectomy (CT) was performed [9]. Modified lateral lymph node dissection (LLND), including levels II–V, was performed in cases with pathologically proven lymph node metastasis (LNM) or suspicious lymph nodes observed intraoperatively or on preoperative imaging.

Suppressive treatment with thyroid hormone was initiated after surgery to decrease serum thyroid-stimulating hormone (TSH) to subnormal levels without clinical thyrotoxicosis. Because its use is strictly controlled in China, radioactive iodine (RAI) therapy was not routinely prescribed for PTC patients after surgery unless patients had distant metastases [9].

### Clinicopathological variable assessment and statistical analysis

Multifocality was defined as 2 or more tumor foci within the thyroid. For multifocal tumors, the diameter of the largest tumor focus was taken as the primary tumor. In addition, data on patient clinical features (gender, age at diagnosis), tumor histological characteristics (maximum size of tumor, extrathyroidal extension), and cervical LNM were extracted from the database.

### Follow-up

All patients gave consent to participate in the follow-up study. The follow-up period for each patient was defined as the length of time from the initial therapy until the last known date of contact documented by a review of the medical record or as a follow-up phone call to the patient. Postsurgical physical examinations were performed every 3–6 months. During the follow-up visits, all patients underwent US examinations of the neck. Recurrence was defined as the appearance of disease, including new biopsy-proven/secondary surgery-confirmed local disease or distant disease revealed by ultrasonography and/or imaging scans, in any patient who had been free of disease (i.e., no palpable disease and negative radioactive iodine scan). New biopsy-proven/secondary surgery-confirmed local disease within the residual thyroid gland or lateral lymph nodes was classified as neck recurrence; distant disease revealed by ultrasonography and/or imaging scans of other sites, including the lungs, bones or brain, was classified as distant recurrence.

### Statistical analysis

The results are expressed as the mean ± standard deviation. Statistical analysis was performed using Student’s *t* test, Χ^2^ test or Mann–Whitney test as appropriate. Patients who were alive and who did not relapse were censored at the date of their last follow-up visit. Neck/metastasis recurrence-free survival (RFS) was defined as the time between the date of initial surgery and the first event of recurrence or death. Overall survival (OS) was defined as the time between the date of initial surgery and death (all causes or cancer-specific). Cancer-specific survival (CSS) was defined as the time between the date of initial surgery and cancer-specific death. Survival rates were estimated using the Kaplan-Meier method [10]. The hazard ratio (HR) and the 95% confidence interval (CI) for relationships between each variable and recurrence were calculated using a binary Cox regression model [11]. A *p* < 0.05 was considered statistically significant. Statistical analyses were performed using SPSS for Windows 13.0 (SPSS Inc., Chicago, IL).

## Results

### Baseline characteristics of patients

At the time of diagnosis, the 496 patients ranged in age from 7 to 85 yr (mean 43.8 ± 17.3 yr). The series comprised 336 females (67.7%) and 160 males (32.3%), with a female/male ratio of 2.1/1. Based on postoperative pathology, the mean maximal tumor size was 2.31 ± 1.59 cm. Multifocality was observed in 209 patients (42.1%). Extrathyroidal extension was evident in 207 cases (41.7%). A total of 381 patients (76.8%) exhibited central lymph node metastasis (CLNM), and 306 patients (61.7%) exhibited lateral lymph node metastasis (LLNM). No patients had a history of familial PTC. The characteristics of the patients are presented in Table 1.

**Table 1:** **Clinicopathological characteristics of 496 papillary thyroid cancer patients**

Variables	Number (%)
Male gender	160 (32.3)
Age	43.8 ± 17.3 yr
Maximum size of tumor (cm)	2.31 ± 1.59
Multifocality	209 (42.1)
Number of tumor foci	1.2 ± 1.3
Extrathyroidal extension	207 (41.7)
Central lymph node metastasis	381 (76.8)
Lateral lymph node metastasis	306 (61.7)

Data are presented as n (%) or mean ± standard deviation.

### Comparison among groups according to the number of tumor foci

Based on the number of tumor foci, the population was divided into 3 groups: G1 (1 tumor focus), G2 (2 foci), and G3 (3 or more foci). Patients with a solitary tumor (n = 287, 57.9%) were classified as G1. Patients with multifocal tumors were classified as G2 (n = 141, 28.4%) or G3 (n = 68, 13.7%). Table 2 presents the characteristics of patients according to the number of tumor foci.

**Table 2:** **Clinicopathological features of papillary thyroid cancer patients according to the number of tumor Foci**

Variables	G1 (n = 287)	G2 (n = 141)	G3 (n = 68)	*p*value
Male gender	94 (32.8)	43 (30.5)	23 (33.8)	NS
Age	47.1 ± 16.1 yr	41.1 ± 18.4 yr	35.5 ± 15.9 yr	<0.05
Maximum size of tumor (cm)	2.29 ± 1.41	2.35 ± 1.9	2.37 ± 1.60	NS
Extrathyroidal extension	115 (40.1)	60 (42.6)	32 (47.1)	NS
Central lymph node metastasis	215 (74.9)	110 (78.0)	56 (82.4)	NS
Lateral lymph node metastasis	173 (60.3)	80 (56.7)	53 (77.9)	NS
Neck recurrence	19 (6.6)	24 (17.6)	14 (20.6)	<0.05
Distant recurrence	8 (2.8)	7 (5.0)	7 (10.3)	NS
Died of thyroid cancer	4 (1.4)	6 (6.4)	5 (7.4)	NS

Data are presented as n (%) or mean ± standard deviation.

*Abbreviations*: *G1* 1 tumor focus, *G2* 2 tumor foci, *G3* 3 or more tumor foci, *NS* not significant.

Age at diagnosis and primary tumor size were compared using the Kruskal-Wallis test.

Group comparisons of categorical variables were performed using the chi-square test or, for small cell values, Fisher’s exact test.

The mean age was 47.1 ± 16.1 yr in G1, 41.1 ± 18.4 yr in G2, and 35.5 ± 15.9 yr in G3 and differed significantly among the 3 groups (*p* = 0.001). A decreasing trend of age was observed from G1 to G3 according to the increasing number of tumor foci.

The incidence of neck recurrence was highest in group G3, followed by group G2 and group G1, and differed significantly among the 3 groups (*p* = 0.037). However, there were no significant differences with respect to gender, maximum tumor size, extrathyroidal extension, CLNM, LLNM, distant recurrence or cancer-specific death among the 3 groups. However, the proportion of aggressive features, such as extrathyroidal extension, CLNM, and LLNM, in the G1 to G3 groups exhibited an increasing trend according to the number of tumor foci.

### Number of tumor foci and recurrence

**Figure 1:**
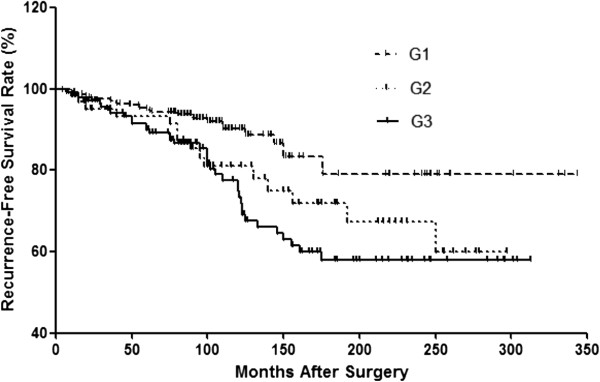
**Kaplan-Meier curves for recurrence-free survival (RFS) in the G1, G2 and G3 groups.** (Log Rank test, *p* = 0.001).

The mean length of follow-up was 124.3 ± 67.8 months, with a range of 10 to 343 months. During the follow-up period, 57 patients (11.5%) experienced neck recurrence, including recurrence in the thyroid or operation bed (n = 13), lateral neck (n = 40), or both locations (n = 4). Distant recurrences occurred in 22 patients (4.4%), including 16 lung metastases, 5 bone metastases, and 1 brain metastasis. Four patients exhibited both neck recurrence and distant recurrence; therefore, the frequency of total recurrence was 15.1% (75/496), and the RFS rate was 84.3% at 10 yr, 70.8% at 15 yr, and 69.6% at 20 yr from the time of the initial operation. The Kaplan–Meier curve was used to investigate the differences in RFS rates among the 3 groups (Figure 1). Patients with 3 or more tumor foci exhibited the shortest RFS, followed by G1 and G2, and differences were significant among the 3 groups (*p* = 0.001, Log Rank test).

To determine how strongly the number of tumor foci was associated with recurrence relative to other known predictors of recurrence in PTC, we performed multivariate Cox regression analysis. Instead of limiting the multivariate analysis to the significant terms from the univariate analysis, we included all variables because these factors have been previously demonstrated to be important in predicting disease recurrence in adult PTC patients. The results are presented in Table 3. The risk of recurrence increased with increasing number of tumor foci, and G3 exhibited a greater risk of recurrence than the G1 group (HR 2.60, 95% CI 1.53-4.39, *p* = 0.001). In addition, extrathyroidal extension was an independent predictor of recurrence (HR 1.95, 95% CI 1.12 -3.38, *p* = 0.018).

**Table 3:** **Multivariate cox regression for recurrence**

Independent variable	Multivariate analysis	
	HR (95% CI)	*p*value
Gender (female *vs.* male)	0.65 (0.41-1.03)	0.063
Age	1.00 (0.99-1.02)	0.872
Size	1.12 (0.98-1.28)	0.108
Extrathyroidal extension	1.95 (1.12 -3.38)	0.018
Number of tumor foci		
G1 (1 tumor focus)	1 (reference)	
G2 (2 foci)	2.06 (1.05-4.01)	0.034
G3 (3 or more foci)	2.60 (1.53-4.39)	0.001
Central lymph node metastasis	0.65 (0.33-1.25)	0.194
Lateral lymph node metastasis	1.07 (0.52-2.16)	0.862

### Number of tumor foci and survival

**Figure 2:**
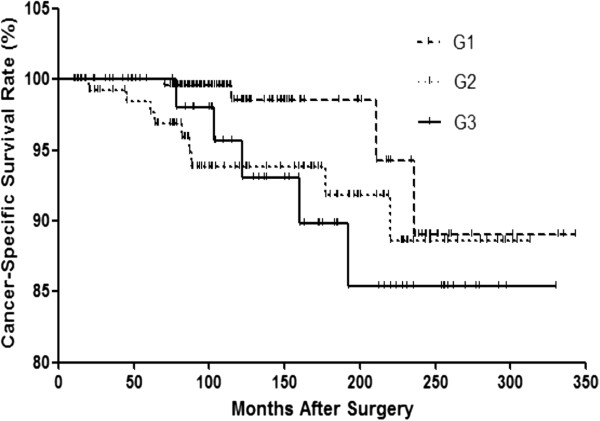
**Kaplan-Meier curves for cancer-specific survival (CSS) in the G1, G2 and G3 groups.** (Log Rank test, *p* = 0.087).

Deaths were observed in 45 patients; however, cancer-specific deaths only occurred in 15 patients. OS and CSS were 92.4% and 96.9% at 10 yr, 86.7% and 94.6% at 15 yr, and 78.6% and 89.1% at 20 yr from the initial operation, respectively. The Kaplan–Meier curve was used to investigate the differences in CSS rates among the 3 groups (Figure 2). The G3 group exhibited the lowest rate of CSS; thus, PTC patients with 3 or more tumor foci had the greatest risk of recurrence, followed by group G2 and group G1. Although a trend of decreasing CSS with increasing number of tumor foci was observed, the differences in CSS were not significant among the 3 groups (*p* = 0.087, Log Rank test).

### Complications

Of the 496 patients, 26 (5.2%) had transient hypoparathyroidism, and 6 (1.2%) had permanent hypoparathyroidism. Recurrent laryngeal nerve injury occurred in 11 patients (2.2%), of which 2 were transient (0.4%) and 9 were permanent (1.8%).

## Discussion

In this study, we investigated the relationship between the number of tumor foci and other clinicopathological features and the impact of multifocality on prognosis in PTC. We observed that an increasing number of tumor foci was associated with both a tendency toward more aggressive features as well as poor prognosis in PTC.

Few studies have addressed the associations between the number of tumor foci and clinicopathological features in multifocal PTCs. Kim *et al.* [12] reported that an increase in the number of tumor foci was strongly associated with older age at diagnosis, cervical LNM, and advanced TNM stage of PTC; furthermore, the number of tumor foci independently predicted LNM. In accordance with their findings, we observed that an increasing number of tumor foci was associated with a tendency toward more aggressive features, such as larger primary tumor size, more frequent extrathyroidal extension and cervical LNM. Although these differences were too small to be significant, they suggest that multifocality might represent the tumor burden and predict more aggressive behavior during disease progression. An interesting finding of the current study was that an increasing number of tumor foci was associated with a younger age at diagnosis in PTC, in contrast to previous reports that advanced age is a prognostic factor for poor prognosis in PTC [12–15]. Younger age may indicate greater risk for the evolution of biological aggressiveness, such as multifocality; we previously reported that younger age is significantly associated with cervical LNM in PTC. However, appropriate initial management may improve the prognosis of younger PTC patients. Younger age as a predictor of multifocality emphasizes the need for more aggressive therapy to achieve the relatively preferable outcomes observed in elderly patients.

Recent studies of the relationship between multifocality and oncological outcomes have suggested that multifocal lesions in PTC are positively associated with poorer prognosis in patients. Kim *et al.* [16] reviewed the medical records of 2095 patients who underwent total thyroidectomy for PTC and reported that multifocality is associated with an increased risk of disease recurrence and persistence, suggesting that the number of tumor foci is a significant predictor of poor clinical outcomes. We determined that multifocality is a risk factor for poor outcomes; furthermore, we observed that a higher number of tumors had a strong linear effect on the risk of recurrence (Table 2 and Figure 1) and was also associated with a trend toward a higher probability for cancer mortality (Table 2), whereas CSS rates did not differ significantly among the 3 groups (Figure 2). In addition, the predictive impact of tumor numbers on recurrence was persistent in multivariate Cox regression analysis when all variables were included (Table 3). Thus, a higher number of tumors, particularly ≥3 tumor foci, is an independent predictor of disease recurrence and a possible indicator of cancer death in PTC. Extrathyroidal extension was another predictor for recurrence, consistent with previous studies [17–19]. However, some studies of the prognostic impact of tumor multifocality in papillary thyroid microcarcinoma (PTMC) have suggested that multifocal lesions do not appear to have prognostic impact in PTMC [20]. Given the high incidence of multifocality in PTMC, 20-40% [21–23], further studies of small PTC tumors are needed to investigate the true role and influence of multifocality in this subtype of PTC.

At our center, TT was not routinely performed in PTC as an initial treatment. Considering the high incidence and negative influence of multifocality, extensive TT surgery is more likely to remove all disease foci and improve prognosis, particularly in PTC patients with ≥3 tumor foci and extrathyroidal extension. In addition, the rates of complications for permanent hypoparathyroidism and recurrent laryngeal nerve injury were both within the ranges of 0-4% and 0-6% reported in previous studies [24, 25], suggesting that thyroidectomy plus lymph node dissections can be performed safely and with low morbidity by experts.

Our study has several limitations that must be taken into account. First, since this study was a retrospective analysis, the prognostic significance of tumor foci has not been fully investigated. The long-term follow-up studies are needed to confirm the prognostic significance in PTC. Second, our study population was a cohort of patients cared for in a single center. Therefore, a much larger number of subjects in multicenter will be needed to generalize this results. At last, the fact that patients in China only receive radioiodine for distant metastases may significantly alter the outcome of patients in other countries where radioiodine is given, but this study certainly shows the impact of significant sized multifocality when radioiodine is not given.

## Conclusion

We observed that a higher number of PTC foci was associated with a tendency toward more aggressive features, including greater primary tumor size, more frequent extrathyroidal extension and cervical LNM. In addition, an increase in the number of tumors was associated with an increased risk of recurrence and a trend toward more cancer mortality. Our findings suggested that the number of tumor foci could be used to assess the risk of poor prognosis, and TT is recommended in patients with more tumor foci.

## Abbreviations

PTC: Papillary thyroid cancer
LNM: Lymph node metastasis
CLNM: Central lymph node metastasis
LLNM: Lateral lymph node metastasis
TT: Total thyroidectomy
CLND: Central lymph node dissection
LLND: Lateral lymph node dissection
CSS: Cancer-specific survival
RFS: Recurrence-free survival
US: Ultrasonography
FNA: Fine-needle aspiration
FS: Frozen sections
TSH: Thyroid-stimulating hormone
RAI: Radioactive iodine.
